# Data survey on the factors affecting students’ satisfaction and academic performance among private universities in Vietnam

**DOI:** 10.1016/j.dib.2020.106357

**Published:** 2020-10-06

**Authors:** Tien Dinh Van, Kim Chi Nguyen Thi, Hong Phuong Tran Thi

**Affiliations:** Hanoi University of Business and Technology, Viet Nam

**Keywords:** Student satisfaction, Academic performance, Private universities, Education program, Training environment

## Abstract

This paper presents the dataset that estimate the effect of factors on students’ satisfaction and their academic performance. The questionnaire with a five-Likert scale ware adapted and developed from prior researches. The sample consisted of 430 fulfilled respondents using stratified random sampling, which recruited from eight private universities in the North of Vietnam. A quantitative method was employed to examine the data. Cronbach's Alpha, Exploratory Factor Analysis, Confirmatory Factor Analysis were utilized to test the reliability and validity of each variable as well as the model fit. Then, the structural equation modeling was used to estimate path coefficients, which can serve as a good reference for further researches.

## Specifications Table

**Table:** 

Subject	Social sciences
Specific subject area	Education
Type of data	Tables and figures
How data were acquired	Survey with questionnaire
Data format	Raw and analysed statistical data
Parameters for data collection	Participants who are full-time students at private universities in Vietnam decided to take part in the survey voluntarily.
Description of data collection	Data were collected by stratified random sampling and based on Internet platforms. The survey were designed by Google Form and the questionnaire was distributed to students who are studying at private universities in Vietnam. The data set consisted of 430 valid responses.
Data source location	City/Town/Region: Private universities, which located in the North of Vietnam. Country: Vietnam Latitude and longitude (and GPS coordinates, if possible) for collected samples/data: 21.028511, 105.804817; 21.18608, 106.07631; 20.959902, 107.042542.
Data accessibility	Data are included in this article. https://data.mendeley.com/datasets/hmp8vbyw5m/draft?a=6bfc6425-332e-414a-9899-762aa8802d0e

## Value of the Data

• This data reflects the satisfaction and academic performance among students at private universities in Vietnam.
• This data presents useful information on key factors related to student satisfaction and their academic performance.
• This data can be served as a reference source for researchers who are interested in the educational sector.
• Examining the influence of factors, including education program, quality of academic staff, service accessibility, training environment and university facilities, on student satisfaction and academic performance not only enables universities and educational administrators to have better solutions to boost the satisfaction and academic performance among students, but it can also help policymakers to propose the appropriate policy to enhance the educational quality of universities.

## 1 Data Description

Student satisfaction and academic performance has been a main focus of both scholars and policymakers in the competitive studying environment [1,2]. Surveying student perception is also considered the most common approach to examine and improve the educational quality of universities [3]. Thus, the vital role of factors such as education program, quality of academic staff, service accessibility, training environment and university facilities in shaping student satisfaction and their academic performance is interested and acknowledged in the education literature [4]. However, there are limited datasets of primary data which is available to explore the effect of factors on satisfaction and academic performance among students at universities.

Moreover, the supplementary role of primary data in that case bases on the multi-dimensional nature of student satisfaction, academic performance as well as how to quantify it. Firstly, the dataset aims to provide raw data, which was directly surveyed from students, to estimate their academic performance, satisfaction. Secondly, it aims to provide the statistical evidence on the effect of education program, quality of academic staff, service accessibility, training environment and university facilities on students’ satisfaction and academic performance. In order to reach these objectives. A questionnaire has been developed and administered to students who attended undergraduate programs at private universities in Vietnam. An outline of fundamental insights utilizing descriptive statistics, exploratory factor analysis (EFA), confirmatory factor analysis (CFA), and structural equation modeling (SEM) is represented in following sections.

Section A: Testing the validity and reliability through Cronbach's Alpha, exploratory factor analysis (EFA) and confirmatory factor analysis (CFA).

Table 1 shows that the Cronbach's alpha of all scales are higher than 0.63 with the lowest level reaching 0.852 (Universities Facilities). Also, the factor loading of each variable is over 0.5. It means that the value of the factor loading estimated from latent variables via observed items and reliability coefficient.

**Table 1:** The results of Cronbach's alpha and exploratory factor analysis (*N* = 430).

Variable	Cronbach's Alpha (α)	Factor loading (λ_i_)					
		(1)	(2)	(3)	(4)	(5)	(6)
Education Program (UP)	0.897						
EP1		0.737					
EP2		0.731					
EP3		0.711					
EP4		0.691					
EP5		0.676					
EP6		0.669					
EP7		0.649					
EP8		0.647					
EP9		0.631					
EP10		0.594					
Quality of academic Staff (QS)	0.921						
QS1			0.759				
QS2			0.729				
QS3			0.681				
QS4			0.672				
QS5			0.653				
QS6			0.632				
QS7			0.631				
QS8			0.580				
QS9			0.550				
QS10			0.542				
Service Accessibility	0.907						
SA1				0.769			
SA2				0.750			
SA3				0.720			
SA4				0.718			
SA5				0.718			
SA6				0.621			
Training Environment	0.896						
TE1					0.756		
TE2					0.745		
TE3					0.720		
TE4					0.718		
TE5					0.616		
TE6					0.585		
University Facilities	0.852						
UF1						0.749	
UF2						0.721	
UF3						0.706	
UF4						0.655	
UF5						0.649	
Academic Performance	0.864						
AP1							0.794
AP2							0.749
AP3							0.737
AP4							0.673
Kaiser–Meyer–Olkin Measure of Sampling Adequacy (KMO)				0.944			
Sig. Of Bartlett's Test of Sphericity				0.000			
Cumulative (%)				62.105			

**Fig. 1:**
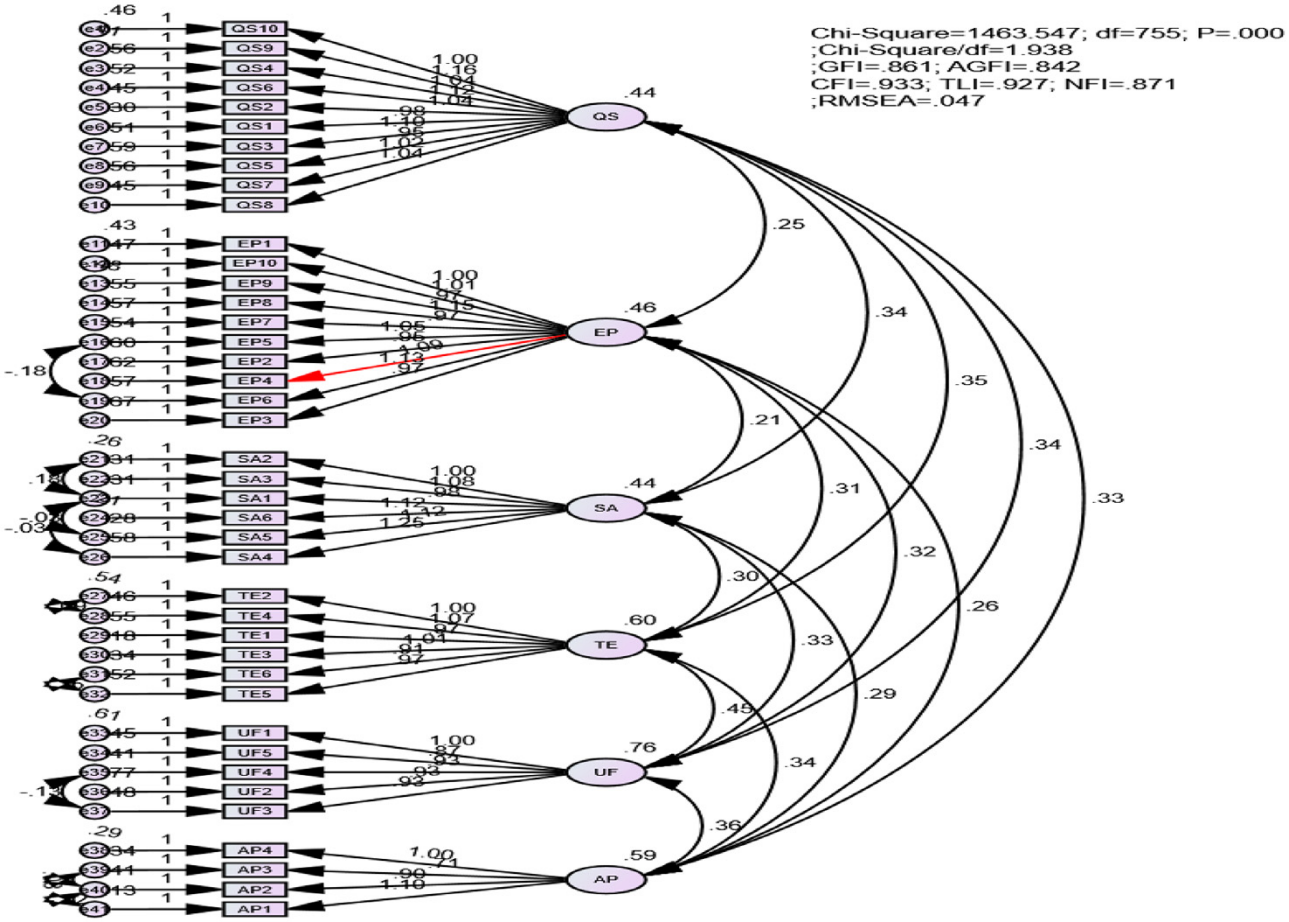
The result of Confirmatory Factor Analysis.

The results of model fit test via using Chi-square, CMIN/DF, GFI, AGFI, CFI, TLI, GFI and RMSEA are summarized in Fig. 1. Although GFI = 0.861 and AGFI = 0.842, almost other values ≥ 0.9. Thus, the model fit is satisfactory, the validity and reliability of all variables are reached [6].

Section B: path coefficients estimated through structural equation modeling (SEM).

**Fig. 2:**
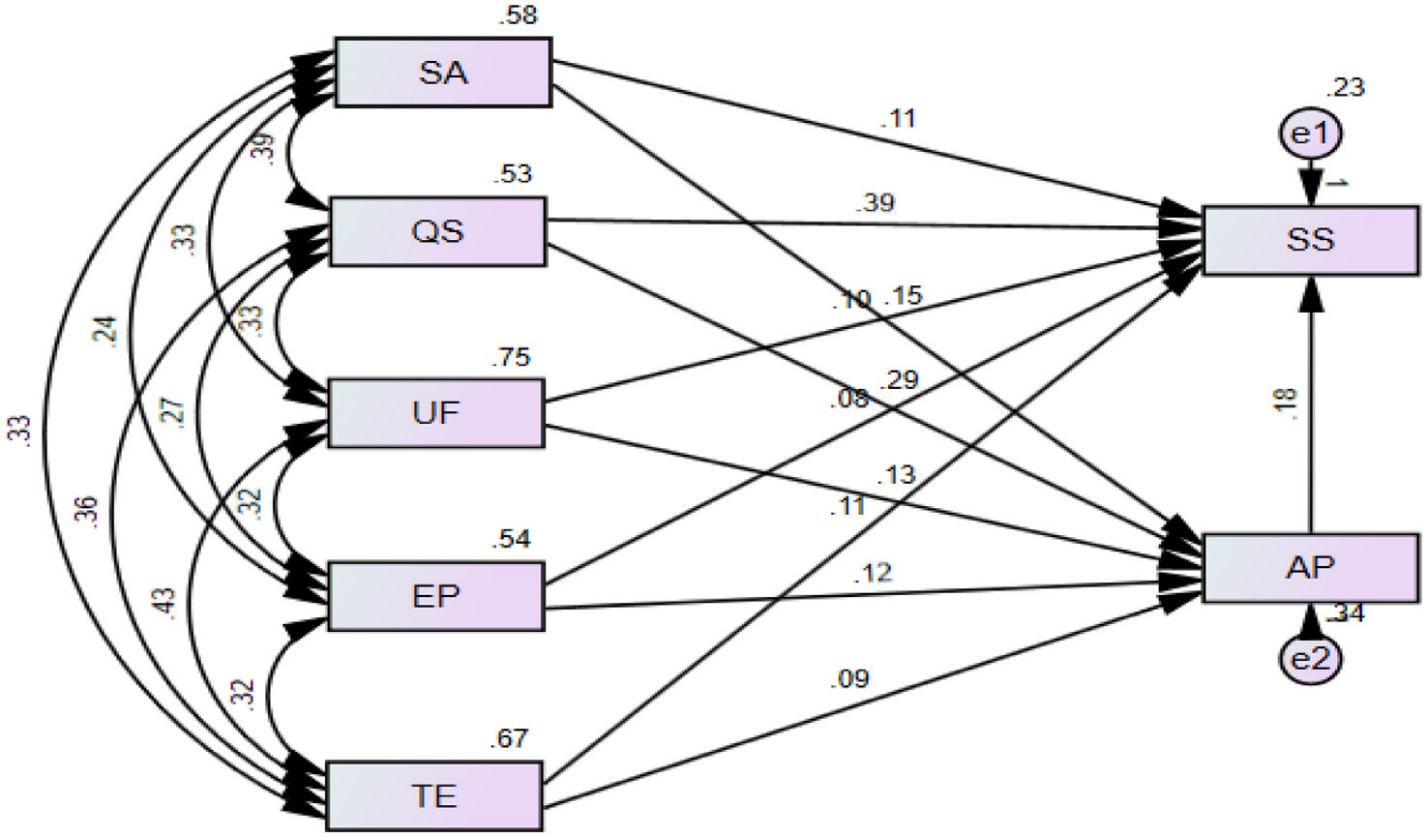
Measurement and structural equation model.

The result of structural equation modeling (SEM) is represented in Fig. 2, while Table 2 describes the regression weights, which can be utilized to examine the linkage between statistical variables in the structural model. Results indicates that quality of academic staff has the strongest effect on students’ academic performance (β = 0.286; *p*-value < 0.001), followed by service accessibility (β = 0.153; *p*-value < 0.01), university facilities (β = 0.126; *p*-value < 0.01), and education program (β = 0.112; *p*-value < 0.05). However, students’ academic performance is not related to training environment (*p*-value > 0.05).

**Table 2:** Path coefficients and Regression weights.

Path coefficients			Estimate	S.E.	C.R.	*P*-value
Service Accessibility	→	Academic performance	0.153	0.054	2.863	0.004
Quality of academic Staff	→	Academic performance	0.286	0.060	4.776	⁎⁎⁎
University Facilities	→	Academic performance	0.126	0.044	2.886	0.004
Education Program	→	Academic performance	0.122	0.048	2.526	0.012
Training Environment	→	Academic performance	0.094	0.049	1.892	0.058
Academic performance	→	Student Satisfaction	0.114	0.044	2.609	0.009
Service Accessibility	→	Student Satisfaction	0.180	0.039	4.590	⁎⁎⁎
Quality of academic Staff	→	Student Satisfaction	0.389	0.050	7.813	⁎⁎⁎
University Facilities	→	Student Satisfaction	0.099	0.036	2.764	0.006
Education Program	→	Student Satisfaction	0.081	0.039	2.069	0.039
Training Environment	→	Student Satisfaction	0.109	0.040	2.699	0.007

Note: *N* = 430,.

⁎⁎⁎ *<* 0.001.

Also, student satisfaction is most strongly affected by quality of academic staff (β = 0.389; *p*-value < 0.001). Service accessibility (β = 0.180; *p*-value < 0.01), academic performance (β = 0.114; *p*-value < 0.01), university facilities (β = 0.099; *p*-value < 0.01), education program (β = 0.081; *p*-value < 0.05), and training environment (β = 0.109; *p*-value < 0.01) is positively related to student satisfaction.

1000 bootstrap samples with a confident degree of 90% is utilized to estimate indirect paths. Table 3 presents direct, indirect and total impacts of factors on student satisfaction. Results show that all linkages between service accessibility, quality of academic staff, university facilities, education program, training environment and student satisfaction are mediated by academic performance.

**Table 3:** The results of direct, indirect and total effect.

			Direct effect		Indirect effect (AP mediator)		Total effects	
			
Path			Estimate	*P*-value	Estimate	*P*-value	Estimate	*P*-value
Service Accessibility	→	Student Satisfaction	0.180	⁎⁎⁎	0.017	0.030	0.197	0.002
Quality of academic Staff	→	Student Satisfaction	0.389	⁎⁎⁎	0.022	0.010	0.441	0.003
University Facilities	→	Student Satisfaction	0.099	0.006	0.023	0.001	0.122	0.002
Education Program	→	Student Satisfaction	0.081	0.039	0.051	0.002	0.132	0.001
Training Environment	→	Student Satisfaction	0.109	0.007	0.028	0.004	0.137	0.009
Academic performance	→	Student Satisfaction	0.114	0.009	–	–	0.114	0.009

Note: *N* = 430.

⁎⁎⁎ *<* 0.001.

## 2 Experimental Design, Materials and Methods

The questionnaire has already been adapted and developed from previous researches [5]. The questions were rated in a 5 Likert-type format from strongly disagree (1) to strongly agree. The survey was performed through the second semester of the academic year 2019–2020. The sample included a total number of 430 students recruited from 8 private universities utilizing stratified random sampling with three-phase procedure. Firstly, eight private universities, including Hanoi University of Business and Technology (HUBT), Phuong Dong University (PDU), Phenikaa University (PU), Thang Long University (TLU), Dai Nam University (DNU), University of Technology and Management (UTM), FPT University (FPT), and Thanh Do University (TDU) were randomly selected from twenty-six ones located in the North of Vietnam. Secondly, five classes at each private university were randomly sampled that based on their studying field. Finally, the research directly distributed the questionnaires to emails of participants at these classes with the supports of teachers. Students were informed that they can take part in the survey voluntarily and their information will be secure and only utilize for the research purpose. Although the sample size only accounted for 430 respondents, however, that is enough for structure equation modeling and the stratified random sampling approach can improve the confidentiality and representativity of the sample [6].

The quantitative analysis was conducted to analyze the data. Particularly, the Cronbach's alpha, exploratory factor analysis (EFA), and confirmatory factor analysis (CFA) were utilized to examine the internal reliability and validity of each scales, then structural equation modeling (SEM) was conducted to explore path coefficients, which was seen as the most appropriate and efficient estimation of the methods for multiple regression analysis. Data is processed using SPSS 23.0 and AMOS 23.0.

## Ethics Statement

The authors received informed consent from participants. Participant was voluntary, and they could withdraw from the survey at any point. As an ethical research team, we value the privacy rights of human subjects. Therefore, the data we submitted does not identify participants based on their responses. The online survey was completely anonymous and does not contain any information allowing identifying the participants.

## Declaration of Competing Interest

The authors declare that they have no know competing for financial interests or personal relationships that could have appeared to affect the work reported in this paper.

## Appendix Supplementary materials

Image, application 1

Image, application 2
