# Supplementary material for: Data survey on the factors affecting students’ satisfaction and academic performance among private universities in Vietnam
Source: Data Brief. 2020 Oct 6;33:106357. doi: 10.1016/j.dib.2020.106357 (PMC7567913; doi:10.1016/j.dib.2020.106357)
Supplement: Supplementary file 1 [file mmc1.docx]

**STUDENTS’ QUESTIONNAIRE**

**Date:**

**No:**

| ***Dear Students!***  ***In order to provide effective solutions to promote school activities, contribute to improving the quality of training services, we look forward to receiving your answers to the following questions. All comments are only for research purposes and will be analysed and interpreted according to the principle of anonymity.***  ***Sincerely thank you!*** |
| --- |

1. **PERSONAL INFORMATION**
2. **Year of study:** ❒ First year ❒Second year ❒Third year ❒Fourth year
3. **Gender:** ❒Male ❒Female
4. **What major do you study?**  …………………………………………………………………………..
5. **ASSESSMENT OF THE SCHOOL TRAINING SERVICE QUALITY**
6. **Please rate your satisfaction with the following contents** (satisfaction levels increase from 1 to 5)

|  | **Very dissatisfied (1)** | **Dissatisfied (2)** | **Confused (3)** | **Satisfied (4)** | **Very**  **satisfied (5)** |
| --- | --- | --- | --- | --- | --- |
| **About accessing educational services of the university** |  |  |  |  |  |
| 1. Academic staffs are never too busy to respond my request for assistance |  |  |  |  |  |
| 1. Academic staffs are sufficient time for consultation |  |  |  |  |  |
| 1. The school's administrative procedures (admission procedures, subject registration ...) of the school in accordance with the process, quickly and promptly |  |  |  |  |  |
| 1. The staffs ensure that they are easily contacted |  |  |  |  |  |
| 1. Information on the website of the university is diversified, and regularly updated |  |  |  |  |  |
| **About the staff, lectures and service staff** |  |  |  |  |  |
| 1. Instructors have the broaden knowledge to answer my questions related to the course content |  |  |  |  |  |
| 1. Instructors deal with courteous manner |  |  |  |  |  |
| 1. When I have a problem, instructors show a sincere interest in solving it |  |  |  |  |  |
| 1. Instructors show positive attitude towards students |  |  |  |  |  |
| 1. Instructors communicate well in classroom |  |  |  |  |  |
| 1. Instructors provide feedback about my progress |  |  |  |  |  |
| 1. Instructors are highly educated in the respective field |  |  |  |  |  |
| 1. The handouts are provided adequately by the instructors |  |  |  |  |  |
| 1. The documentations are provided adequately by the instructors |  |  |  |  |  |
| 1. Supervisors regularly advise, guide, and help students learn and practice |  |  |  |  |  |
| **About School Facilities** | **1** | **2** | **3** | **4** | **5** |
| 1. The system of classrooms, practice rooms, function rooms meets the learning needs of students |  |  |  |  |  |
| 1. Sports and cultural area |  |  |  |  |  |
| 1. Food and beverage service area |  |  |  |  |  |
| 1. Information technology application (computers, internet...) in the school's training |  |  |  |  |  |
| 1. Library (number of seats, number of books, quality of books, references, hours of service ...) |  |  |  |  |  |
| **About the Training / Educational Environment** | **1** | **2** | **3** | **4** | **5** |
| 1. Natural environment (air, trees...) |  |  |  |  |  |
| 1. Many extracurricular activities (culture, sports...) |  |  |  |  |  |
| 1. The attitude of lecturers in training activities (enthusiasm, willingness to support students) |  |  |  |  |  |
| 1. Learning environment, have opportunities to communicate in English |  |  |  |  |  |
| 1. The attitude of administrative staff (training management department, finance, student management...) |  |  |  |  |  |
| 1. The way school leaders receive feedback from students |  |  |  |  |  |
| **About the Study Program** | **1** | **2** | **3** | **4** | **5** |
| 1. The university runs excellent quality program |  |  |  |  |  |
| 1. The university offers a wide range of program with various specializations |  |  |  |  |  |
| 1. The university operates an excellent counselling service |  |  |  |  |  |
| 1. The university offers programs with flexible structure |  |  |  |  |  |
| 1. Contents of the subjects help students foster a sense of self-awareness in learning, self-research, develop creative thinking, practice skills...) |  |  |  |  |  |
| 1. School provide information about the career and job opportunities (through seminars, seminars with employers, announcements at school or on the website) |  |  |  |  |  |
| 1. The training route is arranged in a reasonable order |  |  |  |  |  |
| 1. Rich, diversified and up-to-date textbooks and learning materials |  |  |  |  |  |
| 1. Checking and evaluation activities at school (exam form and content, testing, classification) |  |  |  |  |  |
| 1. The training program is suitable to the requirements of the society and recruitment units |  |  |  |  |  |
| **About Training Results** | **1** | **2** | **3** | **4** | **5** |
| 1. Personal progress (professional knowledge and skills, career, ethical qualities) through school time |  |  |  |  |  |
| 1. Personal progress in self-study, self-research and creativity skills through school time |  |  |  |  |  |
| 1. The ability to solve problems in practice through school time |  |  |  |  |  |
| 1. The level of progress in communication skills, team work .. |  |  |  |  |  |

1. **In your opinion, what solutions are needed to improve the quality of training?**

*..................................................................................................................................................................*

*..................................................................................................................................................................*

1. **How do you assess the strengths / weaknesses of the school compared to the other?**

*..................................................................................................................................................................*

*..................................................................................................................................................................*

1. **Please rate your overall satisfaction with the training quality of school:**

| **Very dissatisfied** | **Dissatisfied** | **Normal** | **Satisfied** | **Very satisfied** |
| --- | --- | --- | --- | --- |
| **1** | **2** | **3** | **4** | **5** |

***Sincerely thank you for your comments and answers!***
